# Abbreviated quantitative UTE imaging in anterior cruciate ligament reconstruction

**DOI:** 10.1186/s12891-019-2811-x

**Published:** 2019-09-14

**Authors:** Takeshi Fukuda, Kenneth Wengler, Dharmesh Tank, Seth Korbin, James M. Paci, David E. Komatsu, Megan Paulus, Mingqian Huang, Elaine Gould, Mark E. Schweitzer, Xiang He

**Affiliations:** 1 Department of Radiology, Stony Brook University, HSC Level 4, Room 120, Stony Brook, NY 11794 USA; 2 Department of Biomedical Engineering, Stony Brook University, Stony Brook, USA; 3 Department of Orthopaedics, Stony Brook University, Stony Brook, USA

**Keywords:** Ultrashort TE (UTE), Anterior cruciate ligament (ACL), ACL reconstruction, Graft healing, Ligamentization, Tendon-to-bone healing

## Abstract

**Background:**

Existing ultrashort echo time magnetic resonance imaging (UTE MRI) methods require prohibitively long acquisition times (~ 20–40 min) to quantitatively assess the clinically relevant fast decay T_2_* component in ligaments and tendons. The purpose of this study was to evaluate the feasibility and clinical translatability of a novel abbreviated quantitative UTE MRI paradigm for monitoring graft remodeling after anterior cruciate ligament (ACL) reconstruction.

**Methods:**

Eight patients who had Graftlink™ hamstring autograft reconstruction were recruited for this prospective study. A 3D double-echo UTE sequence at 3.0 Tesla was performed at 3- and 6-months post-surgery. An abbreviated UTE MRI paradigm was established based on numerical simulations and in vivo validation from healthy knees. This proposed approach was used to assess the T_2_* for fast decay component (${T}_{2s}^{\ast }$) and bound water signal fraction (*f*_*bw*_) of ACL graft in regions of interest drawn by a radiologist.

**Results:**

Compared to the conventional bi-exponential model, the abbreviated UTE MRI paradigm achieved low relative estimation bias for ${T}_{2s}^{\ast }$ and *f*_*bw*_ over a range of clinically relevant values for ACL grafts. A decrease in ${T}_{2s}^{\ast }$ of the intra-articular graft was observed in 7 of the 8 ACL reconstruction patients from 3- to 6-months (− 0.11 ± 0.16 ms, *P* = 0.10). Increases in ${T}_{2s}^{\ast }$ and *f*_*bw*_ from 3- to 6-months were observed in the tibial intra-bone graft (${\varDelta T}_{2s}^{\ast }$: 0.19 ± 0.18 ms, *P* < 0.05; *Δf*_*bw*_: 4% ± 4%, *P* < 0.05). Lower ${T}_{2s}^{\ast }$ (− 0.09 ± 0.11 ms, *P* < 0.05) was observed at 3-months when comparing the intra-bone graft to the graft/bone interface in the femoral tunnel. The same comparisons at the 6-months also yielded relatively lower ${T}_{2s}^{\ast }$ (− 0.09 ± 0.12 ms, *P* < 0.05).

**Conclusion:**

The proposed abbreviated 3D UTE MRI paradigm is capable of assessing the ACL graft remodeling process in a clinically translatable acquisition time. Longitudinal changes in ${T}_{2s}^{\ast }$ and *f*_*bw*_ of the ACL graft were observed.

## Background

The most common knee injuries that require surgical reconstruction are anterior cruciate ligament (ACL) tears [1, 2]. ACL reconstruction usually uses bone-patellar tendon-bone or hamstring tendon autografts [3]. Tendon grafts are composed of densely packed collagen, proteoglycans and cells, but have a different ultrastructure and biochemical composition compared to native ligaments [4]. After ACL reconstruction, two major graft healing processes occur: “ligamentization” of the intra-articular graft and “tendon-to-bone healing” at the graft/bone interface. Ligamentization is the remodeling of the graft into a tissue similar to native ACL [5]. Tendon-to-bone healing involves the development of *enthesis* within the bone tunnel [6]. Although tendon-to-bone healing is well studied in animal models, the applicability of their findings to human grafts is not well documented [7]. The graft/bone interface is a primary site of weakness during the early postoperative period, which is the critical rehabilitation period for successful outcomes [8]. Due to large individual variations in graft healing and the lack of accurate assessment tools for individualized post-operative rehabilitation guidelines, the risk of re-rupture from overloading an inadequately healed graft ranges from 5 to 25% [9–11]. In this context, the development and validation of robust and objective biomarkers to evaluate the individual graft healing status is critical. These biomarkers may also aid in implementing optimal rehabilitation protocols to reduce the risk of graft re-rupture and to allow for quick return-to-play.

Magnetic resonance imaging (MRI) enables non-invasive evaluation of ACL grafts with high tissue contrast, and has been widely used for detecting impingement and graft tears after ACL reconstruction [12]. However, conventional MRI techniques are very limited regarding the graft healing process. Contrast enhanced and diffusion tensor imaging (DTI) MRI have been utilized to evaluate the revascularization and collagen remodeling of implanted grafts, respectively [13–15]. Most of these studies were only conducted at a single time point without longitudinal assessment. In connective tissues including tendons and ligaments, ultrashort echo time (UTE), variable TE (vTE) and DTI MRI have demonstrated the existence of multiple pools of tissue water with distinct MRI properties [16–18]. The fast decay short T_2_* (several 100–1000’s μs) component represents water tightly bound to the highly organized collagen structures, while the slow decay long T_2_* (several 10’s ms) component represents free tissue water [16, 19]. Therefore, analysis of the fast decay T_2_* component in ACL graft potentially reflects the underlying process of collagen remodeling during graft healing.

In previous studies of human Achilles tendon and cortical bone with UTE-based bicomponent T_2_* analysis, changes in the fast decay T_2_* component were linked to collagen disorganization and disruption of the structural integrity, while the slow decay long T_2_* component does not consistently correlate with clinical outcome [16, 20, 21]. The major challenge of the clinical application of the existing bi-exponential T_2_* method involves the sampling of the entire T_2_* decay profile with TEs ranging from several 10’s μs to ~ 20 ms [18], which requires multiple UTE acquisitions with a TR of at least 20 ms and a total acquisition time of ~ 40 min. Recently, protocols which take close to 20 min were reported [16, 22, 23] but are still too costly and time consuming, prohibiting routine clinical screening. In addition, ACL reconstruction patients are unlikely to tolerate such long MRI scans, especially for patients with painful complications.

In this study, we proposed and evaluated a novel abbreviated quantitative UTE MRI paradigm to characterize the fast decay short T_2_* (${T}_{2s}^{\ast }$) component during ACL graft remodeling. The accuracy and clinical translatability of the proposed abbreviated UTE paradigm was also evaluated by numerical simulation and in healthy subjects.

## Methods

### Subjects

A total of 8 patients with primary unilateral ACL tears who underwent Graftlink™ HS autograft reconstruction were recruited between 2013 and 2016 following written informed consent for this institutional review board approved prospective study. All surgeries were performed by the same surgeon and the grafts were placed in an anatomic position. Inclusion criteria were: primary unilateral ACL tear; greater than 18 years of age; existence of an MRI compatible construct. To investigate normal graft healing, potential factors which might make the knee unstable were excluded. Exclusion criteria were: concomitant lateral collateral ligament, posterior cruciate ligament, or posterior lateral corner injury; a greater than grade 2 medial cruciate ligament sprain; grade 3 Outerbridge classification changes in 1 or more compartments (as determined by initial arthroscopy); prior surgery (besides ACL reconstruction) to either knee; insufficient ACL in the contralateral knee. An additional 5 healthy subjects were recruited for validation and test-retest study. Images of healthy ACL and patellar tendon were acquired to assess the proposed abbreviated quantitative UTE paradigm.

### MRI acquisition

MRI of the ACL graft was performed on a 3 T mMR PET/MRI (Siemens, Erlangen, Germany) with a 12-channel knee coil. All patients were imaged at 3- and 6-months post-surgery. In addition to conventional T1- and T2-weighted anatomical images, abbreviated UTE images were obtained. Instead of acquiring a complete set of 3D UTE images with large TE coverage for complete bi-exponential analysis, a reduced set of images with minimal TR were acquired to provide sufficient TE coverage to resolve fast decay ${T}_{2s}^{\ast }$ component. The T_2_* for the slow decay component (${T}_{2l}^{\ast }$) can be determined either from a separate conventional multi-echo gradient echo sequence or by adopting a pre-determined value. We further applied a slab selective RF excitation to reduce the field of view (FOV) along the slice-selection direction, thus reducing the number of views, so that each 3D UTE image was acquired within 2.5 min and the total UTE imaging time was below 8 min for clinical applicability. Most importantly, we adopted a baseline-corrected mono-exponential model to quantify ${T}_{2s}^{\ast }$ for the fast decay component and its signal fraction (*f*_*bw*_). Estimation bias of ${T}_{2s}^{\ast }$ and *f*_*bw*_ from this abbreviated model was corrected accordingly based on pre-calculated formulas from numerical simulations.

Our abbreviated UTE images composed of three sets of 3D double-echo UTE images with TE1/TE2 of 0.1/4.0 ms, 0.27/5.0 ms, and 0.5/6.0 ms. A center-out 3D radial projection *k*-space sampling strategy was used. To reduce TE, half Fourier readout was adopted at the first echo, and full Fourier readout was adopted at the second echo. Other imaging parameters were: FOV of 160 × 160 × 80 mm^3^; slab-selective excitation; voxel size of 1 × 1 × 1 mm^3^ with 80 views along *k*_z_ direction; TR of 13 ms; flip angle of 15°; acquisition time of 2.5 mins. Three healthy subjects were recruited to evaluate the test-retest reliability of the abbreviated acquisition protocol. Subjects were scanned, removed from the MRI, and re-scanned ~ 30 min later. In addition, two healthy subjects were recruited to compare the abbreviated acquisition protocol with a full multi-echo UTE acquisition. This consisted of 5 sets of UTE images with TE1/TE2 of 0.1/4.0, 0.27/7.0, 0.5/10.0, 1.0/14.0 and 2.0/20.0 ms. All other parameters were the same except for a TR of 25 ms. A multi-echo gradient echo variable-TE (vTE) sequence [21] was used to quantify the long T_2_* in the two healthy subjects. The sequence parameters for vTE were: 3D-acquisition; FOV of 160 × 105 × 108 mm^3^; matrix of 128 × 84 × 36; slice oversampling of 22.2%; TR of 40 ms; flip angle of 20°; receiver bandwidth of 640 Hz/Px. The total acquisition time was ~ 3 min for TEs of 0.88, 3.24, 5.37, 7.5, 10, 13 and 19 ms.

### Evaluation of the fast decay short T_2_* component

In ACL patients, the data acquired at the three TE1s (0.1, 0.27, and 0.5 ms) were fit with a baseline-corrected mono-exponential model. The constant baseline was estimated as the average signal from data acquired at the three TE2 s (4.0, 5.0, and 6.0 ms). Since the long T_2_* component has a T_2_* value of ~ 20 ms [19, 21], its signal decay during TE1s can be ignored (~ 3% at TE of 0.5 ms), and its contribution to the total signal can be approximated as a constant baseline. As such, the signal decay at the three TE1s can be attributed solely to the short T_2_* component and modeled by a single compartment mono-exponential decay function with a constant baseline. This leads to signification reduction of the total acquisition time to a level well-tolerated by ACL reconstruction patients (~ 10 min). However, this simplified model may lead to substantial parameter estimation bias. The estimation bias of ${T}_{2s}^{\ast }$ and *f*_*bw*_ will be corrected by utilizing results from numerical simulations. To differentiate the result from conventional bi-exponential fitting, apparent fast decay short T_2_* (${T}_{2s, app}^{\ast }$) and its signal fraction (*f*_*bw,app*_) were used to describe the baseline-corrected mono-exponential T_2_* decay of the MRI signal:

1 $$ S(TE)=\frac{baseline}{1-{f}_{bw, app}}{e}^{- TE/{T}_{2s, app}^{\ast }}+ baseline. $$

Numerical simulations and in vivo data from healthy knees were conducted to assess and correct the estimation bias of ${T}_{2s, app}^{\ast }$ and *f*_*bw*, *app*_ using simulated T_2_* decay profiles from the standard bi-exponential model:

2 $$ S(TE)={S}_0\bullet \left[{f}_{bw}\bullet {e}^{- TE/{T}_{2s}^{\ast }}+\left(1-{f}_{bw}\right)\bullet {e}^{- TE/{T}_{2l}^{\ast }}\right], $$

Where *S*_*0*_ is the initial signal; *f*_*bw*_ is the true bound water fraction; ${T}_{2s}^{\ast }$ and ${T}_{2l}^{\ast }$ are the true transverse relaxation time constants for the short and long components respectively. ${T}_{2s}^{\ast }$ varied between 0.5–2 ms and *f*_*bw*_ varied between 0.25–0.95. Meanwhile, ${T}_{2l}^{\ast }$ took values of 15, 20, and 25 ms [19, 21].

### Data analysis

Scanner raw k-space data for control subjects and ACL patients were imported into Matlab (MathWorks Inc., Natick, MA) for image reconstruction and processing. All UTE images were reconstructed using a modified package from the MRI UNBOUND repository (www.ismrm.org/mri_unbound) courtesy of Nick Zwart. Based on UTE data of a spherical phantom acquired at the same scanner, the adaptive gradient-delay compensation algorithm was utilized to calibrate the sample shift along the readout-direction at both echoes [24, 25]. After a standard 3D re-gridding of shift-corrected *k*-space data, reconstructed spatial UTE images acquired by different receiving channels were sum-of-squares combined.

Regional graft healing was assessed by manual placement of regions-of-interest (ROIs) along the ACL graft by an experienced musculoskeletal fellowship trained radiologist with 10 years of experience. The grafts were separated into three regions; femoral bone tunnel, intra-articular, and tibial bone tunnel. For each bone tunnel segment, the graft itself (intra-bone graft) and the interface between the graft and bone tunnel surface (graft/bone interface) were evaluated. The graft/bone interfaces were assessed by growing the intra-bone graft ROIs by 2 mm [13, 26] and removing any voxels contained within the graft ROIs. Since the ACL graft T_2_* decay profile is orientation-dependent (magic angle effect) [27–30], the relative angles between the graft segments and B_0_ field were determined by estimating the line of best fit through the centroid of each slice of the ROI in the x-y plane and calculating the angle between that line and B_0_. The ${T}_{2s, app}^{\ast }$ and *f*_*bw*, *app*_ were estimated by averaging the signal within each ROI and fitting with the proposed method. For ACL reconstruction patients, ${T}_{2s}^{\ast }$ and *f*_*bw*_ were calculated for each ROI after bias correction assuming ${T}_{2l}^{\ast }$ value of 20 ms. Intra-rater reliability was assessed by having the same radiologist place the ROIs a second time. The relative error between ${T}_{2s}^{\ast }$ and *f*_*bw*_ estimated with the two sets of ROIs and the Dice similarity coefficient between the two sets of ROIs were determined [31].

### Statistical analysis

Parameters were tested for normality using the Lilliefors test; all parameters were normally distributed (*P* > 0.05). Two-tailed, paired t-tests were used for within subject comparisons of the ${T}_{2s}^{\ast }$, *f*_*bw*_ and relative angle with respect to B_0_ at 3- and 6-months post-ACL reconstruction surgery and between the 5 ROIs. A *P*-value less than 0.05 was considered statistically significant.

## Results

### Numerical simulations and in vivo validation

**Fig. 1:**
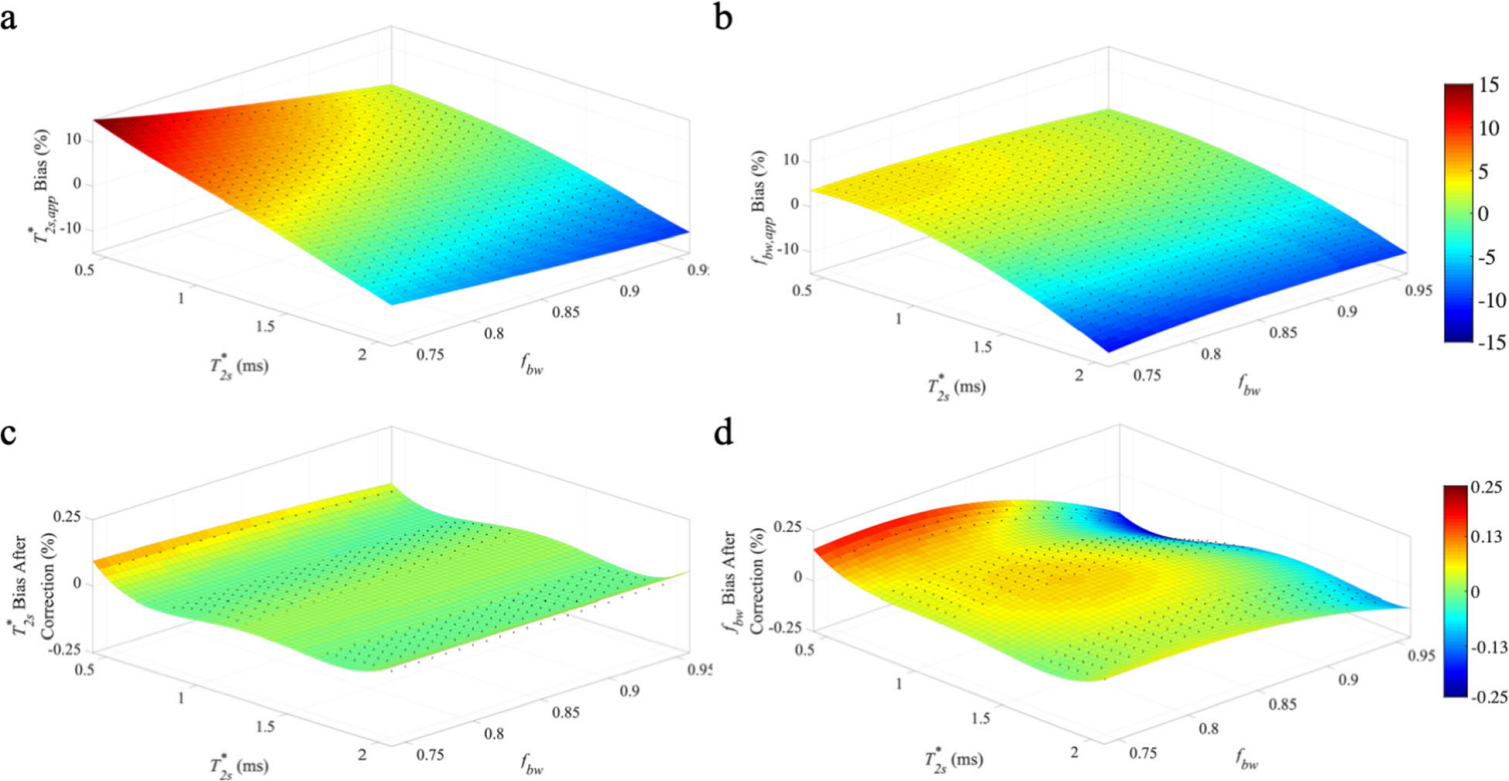
${T}_{2s}^{\ast }$ and *f*_*bw*_ estimation bias over a range of values clinically relevant for ACL grafts. The proposed baseline-corrected mono-exponential model for short T_2_* component evaluation was used for estimation of ${T}_{2s}^{\ast }$ (**c**) and *f*_*bw*_ (**d**). The bias compares the estimated values of ${T}_{2s, app}^{\ast }$ (**a**) and *f*_*bw*, *app*_ (**b**) to the simulated values of ${T}_{2s}^{\ast }$ and *f*_*bw*_. All simulations assumed a ${T}_{2l}^{\ast }$ value of 20 ms

**Fig. 2:**
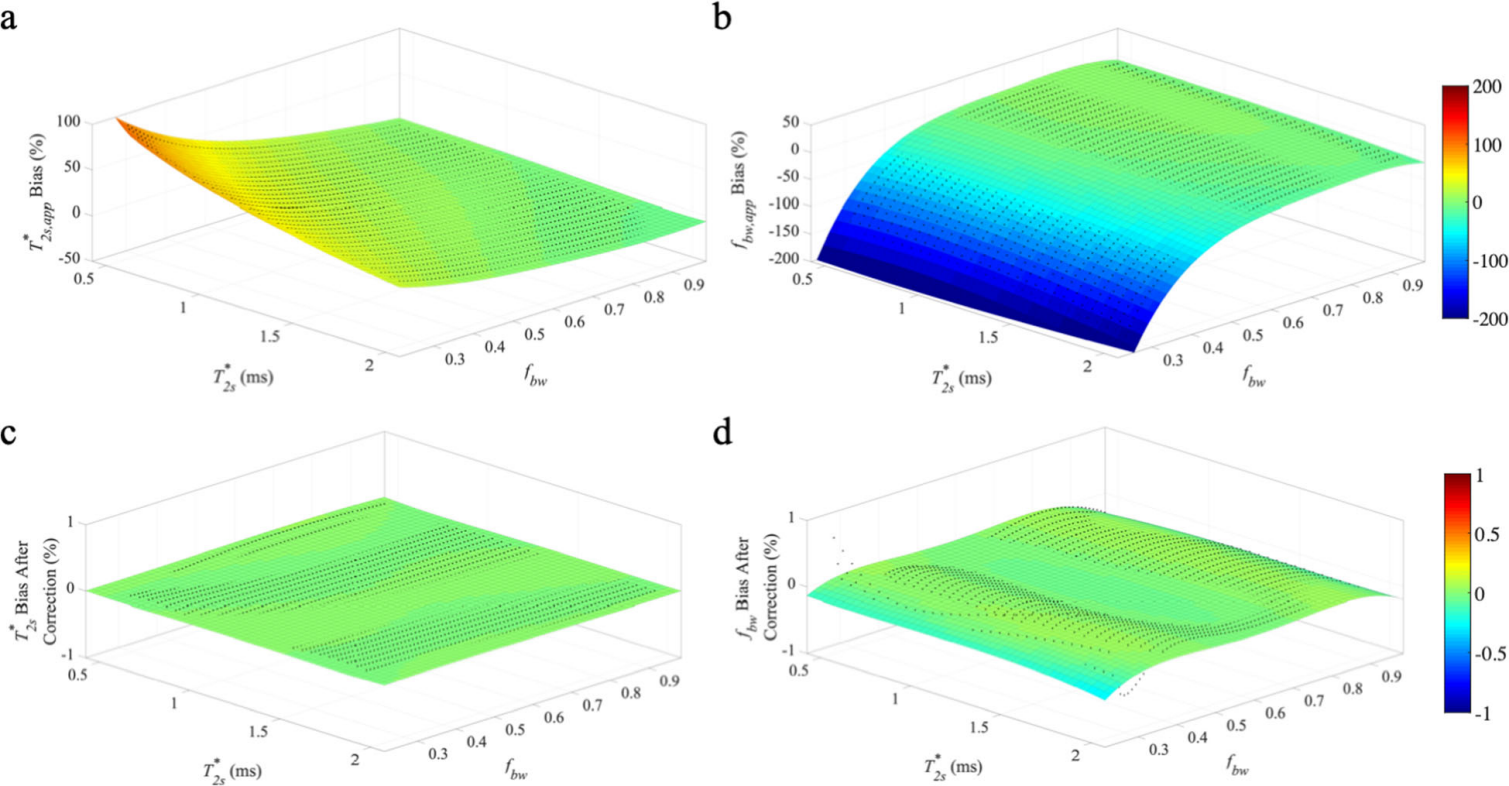
${T}_{2s}^{\ast }$ and *f*_*bw*_ estimation bias over a large range of *f*_*bw*_ values. The proposed baseline-corrected mono-exponential model for short T_2_* component evaluation was used for estimation of ${T}_{2s}^{\ast }$ (**c**) and *f*_*bw*_ (**d**). The bias compares the estimated values of ${T}_{2s, app}^{\ast }$ (**a**) and *f*_*bw*, *app*_ (**b**) to the simulated values of ${T}_{2s}^{\ast }$ and *f*_*bw*_. All simulations assumed a ${T}_{2l}^{\ast }$ value of 20 ms

The estimation bias of ${T}_{2s, app}^{\ast }$ and *f*_*bw*, *app*_ using baseline-corrected mono-exponential model are shown in Fig. 1a and b respectively. Within clinically relevant ${T}_{2s}^{\ast }$ (0.5 ms – 2.0 ms) and *f*_*bw*_ (0.75–0.95) values for ACL graft (with ${T}_{2l}^{\ast }$ of 20 ms), the proposed abbreviated method is relatively robust with low mean relative estimation biases of ±3.7 and ± 3.4% for ${T}_{2s, app}^{\ast }$ and *f*_*bw*, *app*_ respectively. The largest estimation bias for ${T}_{2s, app}^{\ast }$ occurred for small values of ${T}_{2s}^{\ast }$ and *f*_*bw*_ (up to 10% overestimation for ${T}_{2s}^{\ast }$ of 0.5 ms and *f*_*bw*_ of 0.75), and for large values of ${T}_{2s}^{\ast }$ and *f*_*bw*_ (up to 10% underestimation for ${T}_{2s}^{\ast }$ of 2 ms and *f*_*bw*_ of 0.95). The estimation bias for *f*_*bw*, *app*_ was smooth over the range of simulated values that are clinically relevant for ACL grafts; largest estimation bias occurred for large values of ${T}_{2s}^{\ast }$ (up to 14% underestimation for ${T}_{2s}^{\ast }$ of 2 ms and *f*_*bw*_ of 0.75). As shown in Fig. 2, large estimation bias was observed in low *f*_*bw*_ range (0.25–0.75). Over the simulation range of ${T}_{2s}^{\ast }$ (0.5 ms – 2.0 ms) and *f*_*bw*_ (0.25–0.75), with ${T}_{2l}^{\ast }$ of 20 ms, the average relative estimation bias was ±22.9 and ± 42.7% for ${T}_{2s, app}^{\ast }$ and *f*_*bw*, *app*_ respectively.

The formulas for bias correction were derived from polynomial fitting (fourth order) of the estimation error for simulated data with a fixed ${T}_{2l}^{\ast }$ of 20 ms. After bias correction, the average relative estimation error for ${T}_{2s}^{\ast }$ and *f*_*bw*_ was reduced to ±0.01 and ± 0.07% respectively for ${T}_{2l}^{\ast }$ of 20 ms (Fig. 1c and d). Furthermore, the robustness of bias correction formulas (calculated fixed ${T}_{2l}^{\ast }$ of 20 ms) against ${T}_{2l}^{\ast }$ variations was assessed in simulated data with a true ${T}_{2l}^{\ast }$ different than the pre-fixed value of 20 ms. For true ${T}_{2l}^{\ast }$ value of 15 ms, the average ${T}_{2s}^{\ast }$ relative estimation error after bias correction was ±1.0, ±9.4, and ± 7.1% for simulated *f*_*bw*_ values of 0.75–0.95, 0.25–0.75, and 0.25–0.95, respectively. The corresponding average *f*_*bw*_ relative estimation error after bias correction was ±1.4%, ±11.3, and ± 8.5% for simulated *f*_*bw*_ values of 0.75–0.95, 0.25–0.75, and 0.25–0.95, respectively. For true ${T}_{2l}^{\ast }$ value of 25 ms, the average ${T}_{2s}^{\ast }$ relative error after bias correction was ±1.0, ±6.5, and ± 4.9% for simulated *f*_*bw*_ values of 0.75–0.95, 0.25–0.75, and 0.25–0.95, respectively. The corresponding average *f*_*bw*_ relative estimation error after bias correction was ±1.7%, ±6.8, and ± 5.1% for simulated *f*_*bw*_ values of 0.75–0.95, 0.25–0.75, and 0.25–0.95, respectively. ${T}_{2l}^{\ast }$ These results demonstrated that the bias corrected ${T}_{2s}^{\ast }$ and *f*_*bw*_*f*_*bw*_ values showed only limited sensitivity to the exact value of ${T}_{2l}^{\ast }$ for high *f*_*bw*_ values. For low *f*_*bw*_ values, it is necessary to have a separate gradient echo MRI acquisition for accurate quantification of ${T}_{2l}^{\ast }$. The robustness of bias correction formulas was also assessed at different levels of SNR and the resultant estimation error after bias correction was compared to the estimation error of conventional bi-exponential fitting of full UTE data (Table 1). The resultant estimation error of the proposed approach was similar to that of the conventional bi-exponential method.

**Table 1:** Average values of estimation bias of ${T}_{2s}^{\ast }$, and *f*_*bw*_ for abbreviated with proposed bias correction and full UTE over a range of SNR levels. Bias values are averaged over a range of clinically relevant ${T}_{2s}^{\ast }$ (0.5 ms – 2.0 ms) and *f*_*bw*_ (0.70–0.95) values for ACL grafts and a fixed ${T}_{2l}^{\ast }$ value of 20 ms

SNR	Abbreviated UTE		Full UTE	
	*f*_*bw*_ (% error)	${T}_{2s}^{\ast }$ (% error)	*f*_*bw*_ (% error)	${T}_{2s}^{\ast }$ (% error)
1000	0.09 ± 0.07	0.34 ± 0.30	0.10 ± 0.09	0.21 ± 0.16
800	0.12 ± 0.12	0.51 ± 0.45	0.16 ± 0.13	0.31 ± 0.24
600	0.19 ± 0.18	0.90 ± 0.78	0.27 ± 0.23	0.55 ± 0.43
400	0.44 ± 0.46	2.08 ± 1.91	0.61 ± 0.52	1.27 ± 0.95
200	1.96 ± 3.30	9.08 ± 10.2	2.16 ± 1.80	4.99 ± 3.81

**Fig. 3:**
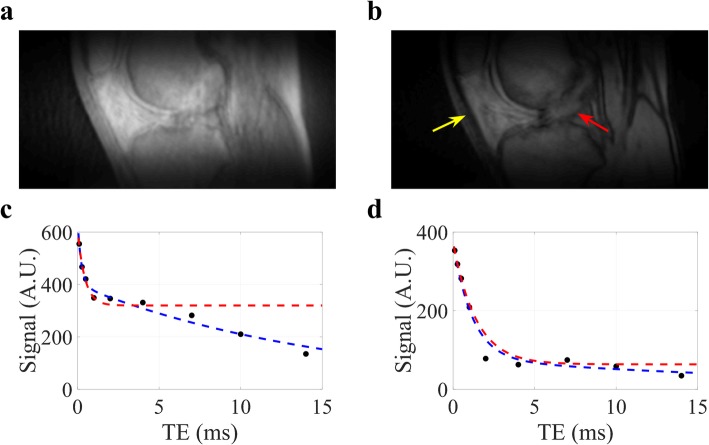
UTE images and signal fitting at TE of 0.1 ms (**a**) and 4 ms (**b**), corresponding signal decay with bi-exponential model fitting (blue lines) and baseline-corrected mono-exponential model fitting (red lines) for the ACL (**c**) and patellar tendon (**d**) for a healthy subject

Figure 3 illustrates UTE images and the corresponding model fitting of the ACL and patellar tendon signal for one of the healthy subjects imaged with 10 TE values. Data from the longest TE (20 ms) was not used because of low SNR. The results from native ACL and patellar tendon of two healthy subjects imaged with both the abbreviated UTE protocol and the full UTE (bi-exponential fitting) protocol are listed in Table 2. As expected, bias correction significantly reduced the estimation bias for ${T}_{2s}^{\ast }$ and *f*_*bw*_ in both ACL and patellar tendon. The estimation bias in healthy ACL is larger than the predicted bias in ACL grafts because of the large contribution (~ 60%) from slow decay ${T}_{2l}^{\ast }$ components in healthy ACL positioned near the magic angle. The relative estimation errors can be further reduced when a patient-specific ${T}_{2l}^{\ast }$ value (based on vTE images) was adopted. For example, the relative errors for ${T}_{2s}^{\ast }$ and *f*_*bw*_ in ACL measurement were reduced from 24 and 7% to 13% and − 6%, respectively, when a vTE-estimated ${T}_{2l}^{\ast }$ value of 14 ms was used for the first subject. Similarly, the relative errors for ${T}_{2s}^{\ast }$ and *f*_*bw*_ in patellar tendon were reduced from 9% and − 7 to 4% and − 2%, respectively, when a vTE-estimated ${T}_{2l}^{\ast }$ value of 29 ms was used for the first subject.

**Table 2:** Average values of ${T}_{2s, app}^{\ast }$, *f*_*bw*, *app*_, ${T}_{2s}^{\ast }$, and *f*_*bw*_ from 2 healthy subjects in validation study

Subject	Patellar Tendon							ACL						
	Abbreviated UTE				Full UTE			Abbreviated UTE				Full UTE		
	*f*_*bw*, *app*_ (%)	${T}_{2s, app}^{\ast }$ (ms)	*f*_*bw*_ (%)	${T}_{2s}^{\ast }$ (ms)	*f*_*bw*_ (%)	${T}_{2s}^{\ast }$ (ms)	${T}_{2l}^{\ast }$ (ms)	*f*_*bw*, *app*_ (%)	${T}_{2s, app}^{\ast }$ (ms)	*f*_*bw*_ (%)	${T}_{2s}^{\ast }$ (ms)	*f*_*bw*_ (%)	${T}_{2s}^{\ast }$ (ms)	${T}_{2l}^{\ast }$ (ms)
1	73.1	1.33	73.8	1.29	79.3	1.18	23.87	21.4	0.60	44.2	0.42	41.3	0.34	16.58
2	82.7	1.07	82.3	1.04	76.7	0.98	20.62	31.8	0.81	48.3	0.63	46.2	0.42	14.40

The simulation and healthy knee results demonstrated that for high *f*_*bw*_ (> 75%), estimation bias for ${T}_{2s, app}^{\ast }$ and *f*_*bw*, *app*_ is relatively small (~ 1–6%) after bias correction adopting a fixed ${T}_{2l}^{\ast }$ value. Conversely, for tissues with low *f*_*bw*_ (< 75%), the relative bias can be high (from ±23 to ±9% for ${T}_{2s}^{\ast }$ and from ±43 to ±10% for *f*_*bw*_) after bias correction with a fixed ${T}_{2l}^{\ast }$ value. To further reduce estimation bias, an additional subject-specific tissue ${T}_{2l}^{\ast }$ value, which can be estimated from conventional multi-echo gradient echo images, should be adopted in bias correction. The test-retest results from 3 healthy volunteer subjects are listed in Table 3. Overall, the proposed method achieved with ~ 1% deviation between the first and second scans for patellar tendon measurements and ~ 5% deviation between the first and second scans for ACL measurements.

**Table 3:** Average values of ${T}_{2s}^{\ast }$ and *f*_*bw*_ from 3 healthy test-retest subjects

Subject	Test				Retest			
	Patellar Tendon		ACL		Patellar Tendon		ACL	
	*f*_*bw*_ (%)	${T}_{2s}^{\ast }$ (ms)	*f*_*bw*_ (%)	${T}_{2s}^{\ast }$ (ms)	*f*_*bw*_ (%)	${T}_{2s}^{\ast }$ (ms)	*f*_*bw*_ (%)	${T}_{2s}^{\ast }$ (ms)
1	85.4	0.80	55.6	0.42	84.4	0.79	57.1	0.40
2	83.3	1.03	61.5	0.40	84.4	1.06	62.9	0.34
3	83.6	0.87	62.6	0.52	83.6	0.87	61.8	0.52

### ACL graft reconstruction

**Fig. 4:**
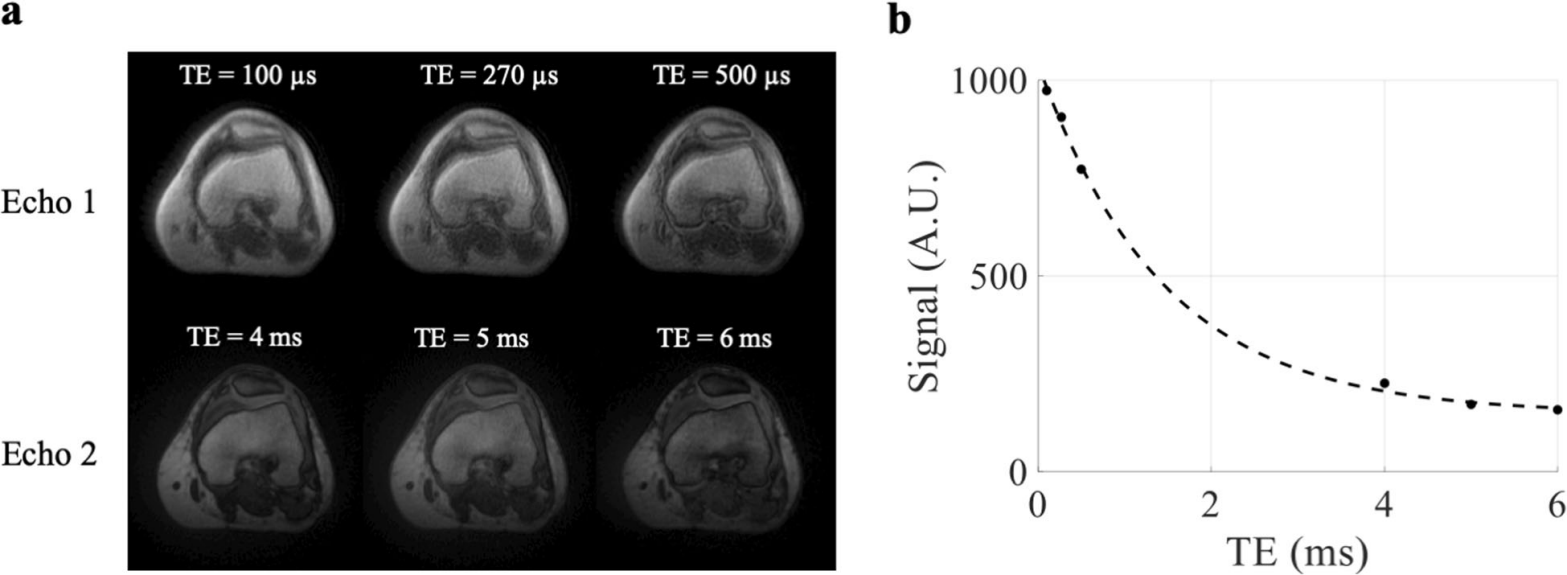
UTE images at the 6 imaged TEs (**a**) and the corresponding signal decay profile with baseline-corrected mono-exponential fitting from the intra-articular graft region (**b**) for a representative ACL reconstruction subject. Note that the TE2 images are scaled by a factor of 1.2 for display purposes

**Fig. 5:**
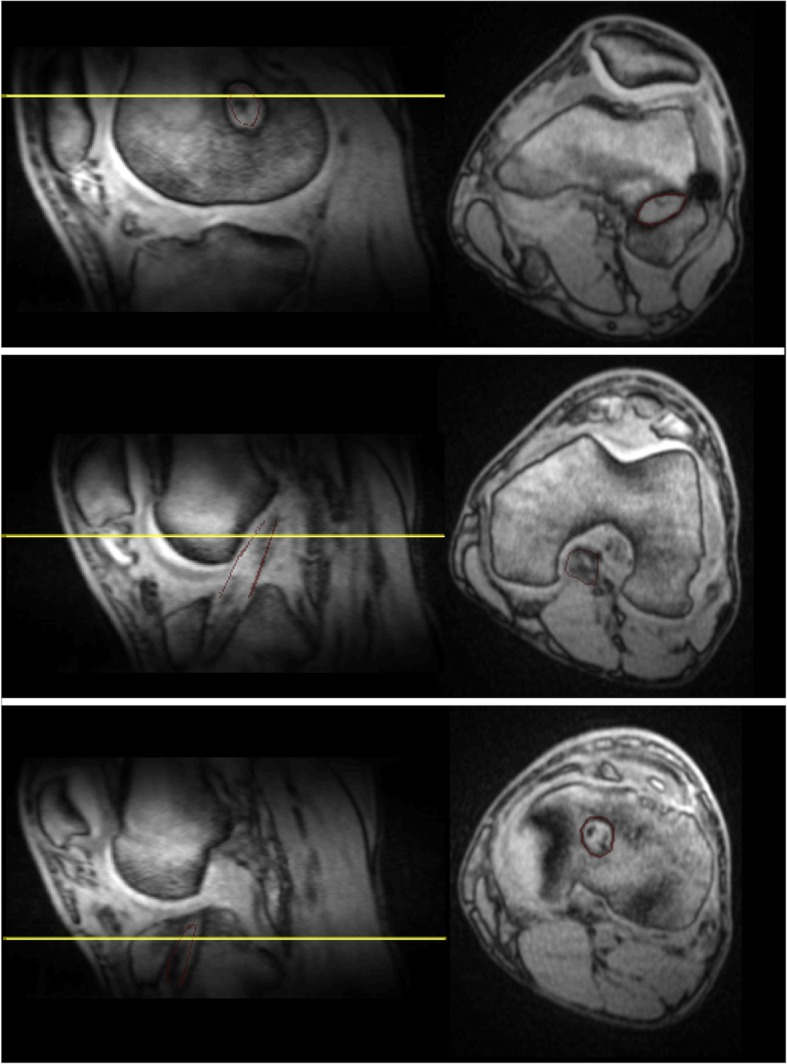
Sagittal and axial UTE image with TE of 4 ms for a representative ACL reconstruction subject. The left column shows the images in the sagittal plane and the right column shows images from the axial planes denoted by the yellow lines in the sagittal plane images. The red lines delineate the ACL graft. The first row depicts the femoral intra-bone graft and graft/bone interface, the second row depicts the intra-articular graft, and the third row depicts the tibial intra-bone graft and graft/bone interface. The outlines of the ACL graft on the sagittal plane images have been shrunk by 2 voxels for illustration purposes. The ACL graft outlines on the axial plane images were not shrunk by 2 voxels. The ACL graft outlines on the axial plane images of the femoral and tibial graft tunnels are thicker than the ACL graft outlines on the axial plane image of the intra-articular graft because they include the graft/bone interface (outer 2 voxels)

**Fig. 6:**
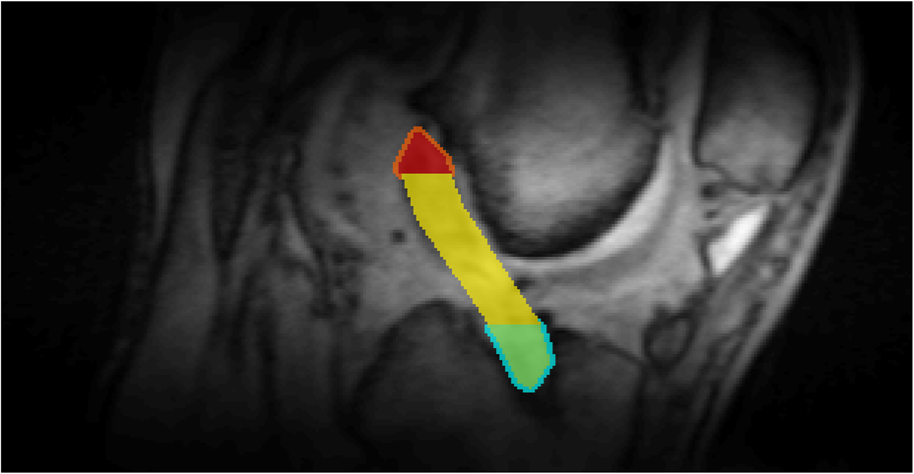
Regions of interest for a typical ACL reconstruction subject overlaid on the UTE image with TE of 4 ms. ROIs are color coded as follows: orange, femoral graft/bone interface; red, femoral intra-bone graft; yellow, intra-articular graft; green, tibial intra-bone; blue, tibial graft/bone interface

**Fig. 7:**
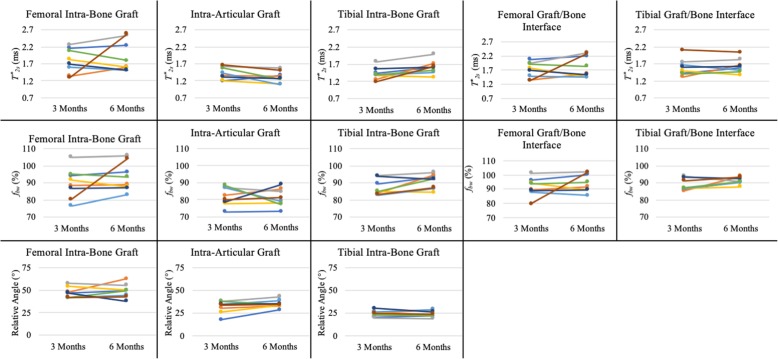
Ladder plots of the ${T}_{2s}^{\ast }$, *f*_*bw*_, and angle relative to B_0_ from 8 ACL reconstruction subjects at 3- and 6-months post-ACL reconstruction surgery

The operated knees of all ACL reconstruction patients were confirmed as stable and no complications were documented clinically. Figure 4a shows the UTE images at the 6 TEs for a representative ACL reconstruction subject. Compared to reconstructed UTE images from TE2 (full Fourier), the TE1 images (half Fourier) demonstrated lower SNR and some streaking artifacts due to reduced k-space sampling density along the *k*_z_ direction. Nevertheless, the UTE images exhibited high contrast between different tissues, allowing for accurate delineation of the ACL graft, as shown in a sagittal and axial view of ACL graft (Fig. 5). The average signal and model fitting from the intra-articular region are displayed in Fig. 4b. The signal from the intra-articular graft demonstrated an exponential behavior over the three shortest TEs; accurate fitting of the data was achieved with ${T}_{2s}^{\ast }$ of 1.40 ms and *f*_*bw*_ of 85%. Figure 6 displays the ROI selection for a typical subject. The ROIs demonstrated varying orientation along the ACL graft; the relative angles with respect to B_0_ in the femoral intra-bone, intra-articular, and tibial intra-bone graft segments were 41.6°, 34.1°, and 26.4° respectively. The *f*_*bw*_ and ${T}_{2s}^{\ast }$ values at 3- and 6-months post-surgery in the 5 ROIs, as well as the relative angle in the 3 graft ROIs, from the 8 ACL reconstruction subjects are shown in Fig. 7, with the average values listed in Table 4. The results of intra-rater reliability are listed in Table 5.

**Table 4:** Average values of ${T}_{2s}^{\ast }$, *f*_*bw*_, and angle relative to B_0_ from all ACL reconstruction patients

Region	3-Months			6-Months		
	*f*_*bw*_ (%)	${T}_{2s}^{\ast }$ (ms)	Angle (°)	*f*_*bw*_ (%)	${T}_{2s}^{\ast }$ (ms)	Angle (°)
Femoral intra-bone graft	90 ± 9	1.78 ± 0.37	47.3 ± 5.9	93 ± 8	1.92 ± 0.46	49.0 ± 7.7
Intra-articular graft	82 ± 5	1.42 ± 0.18	31.6 ± 6.8	81 ± 5	1.31 ± 0.17	35.0 ± 4.3
Tibial intra-bone graft	87 ± 5	1.43 ± 0.18	24.6 ± 3.4	91 ± 4	1.61 ± 0.20	24.5 ± 3.4
Femoral graft/bone interface	91 ± 6	1.69 ± 0.29		94 ± 6	1.83 ± 0.38	
Tibial graft/bone interface	89 ± 3	1.60 ± 0.25		91 ± 2	1.64 ± 0.21

**Table 5:** Intra-rater reliability showing average values of the relative error of ${T}_{2s}^{\ast }$ and *f*_*bw*_ measurements and the Dice similarity coefficient from all ACL reconstruction patients

Region	*f*_*bw*_ (% error)	${T}_{2s}^{\ast }$ (% error)	Dice
Femoral intra-bone graft	0.9 ± 0.9	1.2 ± 1.3	0.93 ± 0.02
Intra-articular graft	1.1 ± 0.8	1.3 ± 1.1	0.89 ± 0.03
Tibial intra-bone graft	1.0 ± 0.8	1.3 ± 1.4	0.91 ± 0.01
Femoral graft/bone interface	1.2 ± 1.2	1.0 ± 0.7	0.90 ± 0.01
Tibial graft/bone interface	1.0 ± 1.1	1.1 ± 1.5	0.91 ± 0.02

### Analysis of intra-articular graft, “ligamentization”

The average ${T}_{2s}^{\ast }$ and *f*_*bw*_ at the 3-month timepoint were 1.42 ± 0.18 ms and 82 ± 5%, respectively. The estimated values at the 6-month timepoint were 1.32 ± 0.15 ms and 79 ± 6%, respectively. A decrease in ${T}_{2s}^{\ast }$ was observed in 7 of the 8 ACL reconstruction patients at the second time point compared to the first (− 0.11 ± 0.16 ms, *P* = 0.10). Meanwhile, almost no change in *f*_*bw*_ (− 1% ± 7%, *P* = 0.76) was observed. A greater angle between the intra-articular graft and B_0_ was observed at the 6-month time point (31.6° ± 6.8° vs. 35.0° ± 4.3°, *P* = 0.07).

### Analysis of intra-bone graft, “graft healing inside of the bone tunnel”

In the femoral intra-bone graft, the average ${T}_{2s}^{\ast }$ and *f*_*bw*_ at the 3-month timepoint were 1.78 ± 0.37 ms and 90% ± 9%, respectively. The corresponding values in the tibial intra-bone graft were 1.43 ± 0.18 ms and 87% ± 5%, respectively. The values at the 6-month time point were 1.92 ± 0.46 ms and 93% ± 8%, respectively for the femoral intra-bone graft, and 1.61 ± 0.20 ms and 91% ± 4%, respectively for the tibial intra-bone graft. Significant increases in ${T}_{2s}^{\ast }$ and *f*_*bw*_ from the 3- to 6-month time point were observed in the tibial intra-bone graft (${T}_{2s}^{\ast }$: 0.14 ± 0.14 ms, *P* < 0.05; *f*_*bw*_: 4% ± 4%, *P* < 0.05). Non-significant increases were observed in the femoral intra-bone graft (${T}_{2s}^{\ast }$: 0.14 ± 0.51 ms, *P* = 0.46; *f*_*bw*_: 4% ± 9%, *P* = 0.29). No differences in the relative angle of the intra-bone grafts and B_0_ were observed between the two time points (47.3° ± 5.9° vs. 49.0° ± 7.7°, *P* = 0.54 for femoral intra-bone graft, and 24.6° ± 3.4° vs. 24.5° ± 3.4°, *P* = 0.97 for tibial intra-bone graft).

### Analysis of graft/bone interface, “tendon-to-bone healing”

At the femoral graft/bone interface, the average ${T}_{2s}^{\ast }$ and *f*_*bw*_ at the 3-month time point were 1.69 ± 0.29 ms and 91% ± 6%, respectively. The corresponding values were 1.60 ± 0.25 ms and 89% ± 3%, respectively at the tibial graft/bone interface. The values at the 6-month time point were 1.83 ± 0.38 ms and 94% ± 6%, respectively at the femoral graft/bone interface, and 1.64 ± 0.21 ms and 91% ± 2%, respectively at the tibial graft/bone interface. Slight, but not significant, increases in ${T}_{2s}^{\ast }$ from the 3- to 6-month time point were observed (femoral graft/bone interface: 0.14 ± 0.40 ms, *P* = 0.37; tibial graft/bone interface: 0.04 ± 0.16 ms, *P* = 0.48). Additionally, non-significant increases in *f*_*bw*_ were observed (femoral graft/bone interface: 3% ± 8%, *P* = 0.34; tibial graft/bone interface: 2% ± 4%, *P* = 0.10).

When comparing the intra-bone graft to the graft/bone interface in the femoral tunnel, *f*_*bw*_ was non-significantly higher (2% ± 4%, *P* = 0.33) and ${T}_{2s}^{\ast }$ was significantly lower (− 0.09 ± 0.11 ms, *P* < 0.05) at the 3-month time point. The same comparisons at the 6-month time point also yielded significantly lower ${T}_{2s}^{\ast }$ (− 0.09 ± 0.12 ms, *P* < 0.05) and no difference in *f*_*bw*_ (1% ± 3%, *P* = 0.30). In the tibial tunnel, no significant differences were observed at the 3-month time point (*f*_*bw*_: 2% ± 3%, *P* = 0.20; ${T}_{2s}^{\ast }$: 0.17 ± 0.31 ms, *P* = 0.16) or the 6-month time point (*f*_*bw*_: 0% ± 4%, *P* = 0.77; ${T}_{2s}^{\ast }$: 0.03 ± 0.17 ms, *P* = 0.63).

## Discussion

ACL graft healing involves dramatic changes to tissue macrostructure and organization, which can affect the transverse relaxation time (T_2_/T_2_*) of collagen bound tissue water. To alleviate the time burden of conventional bi-exponential UTE, we proposed a simplified, abbreviated 3D UTE paradigm with bias correction to monitor the T_2_* dynamics of fast decay component after ACL reconstruction surgery. The feasibility and accuracy of the proposed paradigm was quantitatively assessed by numerical simulations and in vivo data of healthy knees. With less than 8 mins of total MRI acquisition time, a decrease of ${T}_{2s}^{\ast }$ was observed in the intra-articular graft, consistent with the underlying changes of collagen remodeling and reorganization during the ligamentization process. This study demonstrated the clinical translatability of the proposed abbreviated UTE protocol, suggesting the possibility of future applications in other connective tissues.

The major challenge in conducting UTE bi-exponential T2* analysis is the long acquisition time. Kijowski et al. utilized a center-out twisted 3D-Cones k-space sampling scheme for assessing fast T_2_* component in patellar tendinopathy with total scan time of 19 min [16]. A total of 16 echoes ranging from 0.03 to 30.0 ms were acquired. A 3D fat saturated UTE-Cones acquisition scheme with total scan time of 18 min was evaluated for single- and bi-exponential analysis of T_2_* relaxation in knee tendons and ligaments [23]. However, these approaches are still time consuming. Recently the feasibility of using Cartesian sampling with sub-millisecond TE was assessed in menisci. Juras et al. demonstrated that 3D Cartesian variable echo time (vTE) gradient echo may allow for high-resolution quantitative bi-exponential T_2_* analysis in degenerative menisci with total scan time of 12 mins [22]. However, the smallest TE of 0.75 ms limits its accuracy in quantifying fast-decay components in certain connective tissues.

In this study, the proposed abbreviated 3D UTE paradigm utilized several techniques to reduce the total acquisition time and improve the accuracy of ${T}_{2s}^{\ast }$ and *f*_*bw*_ quantification. By adopting a slab-selective half-sinc RF pulse for excitation, the FOV along the slice-selection direction was reduced, thus reducing the number of views acquired along the *k*_z_ direction. While no noticeable imaging artifacts were observed in the reconstructed images at the second echo (TE2), some minor streaking artifacts at the first echo (TE1) can be seen. This was caused by a lower sampling density at TE1 (half Fourier) compared to TE2 (full Fourier). This effect was minimized in our analysis by performing ROI instead of voxel-wise T_2_* quantification. To further reduce the acquisition time, fat suppression was not utilized. This can potentially lead to increased UTE imaging artifacts due to the chemical shift of lipids. With a readout bandwidth of 560 Hz/pixel, the shift is less than one pixel and no noticeable chemical shift artifacts in UTE images were observed. To reduce potential confounds from high intensity lipid signal and for better delineation of the ACL graft, spectral selective lipid suppression techniques can be utilized in future studies [32]. By acquiring only 3 sets of 3D double-echo UTE images with maximum TE of 6.0 ms (to reduce TR and acquisition time), we do not have sufficient TE coverage to conduct complete bi-exponential fitting. As demonstrated by numerical simulations, the accuracy of the ${T}_{2s}^{\ast }$ and *f*_*bw*_ quantification using the abbreviated UTE paradigm is better for lower ${T}_{2s}^{\ast }$ and higher *f*_*bw*_, which is the case for ACL grafts. Large estimation bias in low *f*_*bw*_ range was thought to be due to the violation of the underlying assumption for the baseline-corrected mono-exponential model that the ${T}_{2l}^{\ast }$ compartment contributes minimally to the total UTE signal at TE1s. As demonstrated in this study, the abbreviated UTE acquisition with the chosen 6 TEs allows for accurate estimation of ${T}_{2s}^{\ast }$ and *f*_*bw*_ within a clinically feasible acquisition time of less than 8 min after adopting a similar bias correction approach that was implemented in our recent publication [33]. The abbreviated acquisition does come at the cost of reduced spatial resolution and reduced SNR. The SNR of a single voxel of the shortest TE image for intra-articular ACL graft was ~ 75; while the estimation bias will increase with lower SNR, by averaging ~ 20,000 voxels for ACL graft regions and ~ 5000 voxels for graft/bone interface regions this problem was avoided. As demonstrated in this study in healthy knees, to further improve the accuracy of ${T}_{2s}^{\ast }$ and *f*_*bw*_ estimation especially for tissues with long ${T}_{2s}^{\ast }$ and/or low *f*_*bw*_, bias correction with accurate slow decay component (${T}_{2l}^{\ast }$) characterization should be implemented. This can be directly estimated by acquiring additional conventional multi-echo gradient echo data (~ 3 mins) for improved time efficiency.

After ACL reconstruction, two major graft healing processes occur: “ligamentization” of the intra-articular graft [5, 34] and “tendon-to-bone healing” at the graft/bone interface [6]. Early ligamentization includes the graft healing and proliferation phase, with disintegration of collagen fibrils and increased synthesis of type III collagen occurring during 4–12 weeks post-operation [11, 34]. The ligamentization phase occurs from 3 to 12 months post-operation when the graft reorganizes into densely packed collagen bundles, resembling the appearance of a native, intact ACL [34]. Tendon-to-bone healing at the graft/bone interface is facilitated by fibrovascular tissue and forms perpendicular collagen bundles inserting directly into the bone, similar to Sharpey’s fibers [35, 36] which can be identified around 12–15 weeks post-surgery [6, 37]. The fibers are present until ~ 1 year after surgery, while gradual osseointegration occurs from the bone surface [38]. Due to the difficulty of in vivo access to the interior of the bone tunnel by biopsy or arthroscopy, the intra-bone graft healing process has not been well studied. In this context, the development and validation of robust and objective biomarkers to evaluate the individual ligamentization and tendon-to-bone healing status is necessary for monitoring successful outcomes of ACL reconstruction.

In both Achilles and patellar tendons, increased T_2_* values for the fast decay component were reported following injury [16, 21, 39]. Our results showed a decrease in ${T}_{2s}^{\ast }$ for the intra-articular graft at ~ 6-months compared to ~ 3-months post-operation, which might be consistent with the ligamentization phase. However, the ${T}_{2s}^{\ast }$ values are still elevated compared to that of healthy ACL (less than 1 ms) [20], even at 6-months post-surgery. This could be due to differences in the structure of the regained collagen fibers compared to native ACL or incomplete ligamentization [40–43], since these regained collagen fibers show homogeneous small diameters while original ligaments have a bimodal distribution of small and large collagen fibers [44]. Additionally, a study has suggested that the graft of human subjects undergoes ligamentization at a slower rate than what is reported in animal studies [34]. Hence, more patients and more time points are needed to investigate whether the reconstructed ACL graft will eventually regain the ${T}_{2s}^{\ast }$ of a native ACL, and to better understand the time-course of the human graft ligamentization.

We hypothesize that the establishment of fibro-osseous connections at the graft/bone interface may restrict the collagen bound water and our results showed an increase of *f*. A previous study suggested that grafts inside of the bone tunnel also undergo a remodeling process [26]. Although not validated with graft histology, we observed an increase of ${T}_{2s}^{\ast }$ and *f*_*bw*_ from 3- to 6-months after surgery. This change differed from our observations in the intra-articular graft and may suggest that the intra-bone graft undergoes a different remodeling process. Currently, there is no supporting histological studies exploring the intra-bone graft healing. We speculate that the observed higher ${T}_{2s}^{\ast }$ and *f*_*bw*_ values at intra-bone graft may be caused by the accumulation of the inflammatory cells produced by bone marrow surrounding intra-bone graft, which might be more prominent in intra-bone graft than intra-articular graft. Hence, our results may provide new insight into intra-bone graft healing but further studies with more subjects and more time points are required to better elucidate this healing process.

Tendons and ligaments are known to experience orientation dependent changes to their T_2_* value [27–30]. As such, it is important that whenever T_2_* is measured in these tissues to consider the orientation of the tissue with respect to B_0_. In this study, because subjects were all imaged with a standard knee coil, setup and positioning of the knee was relatively consistent. No differences in orientation of the femoral and tibial intra-bone grafts between the two time points were observed, suggesting that any measured changes in these regions were not affected by the magic angle effect. Conversely, the intra-articular graft was closer to the magic angle at the 6-month time point (~ 35°) than at the 3-month time point (~ 32°), causing the measured ${T}_{2s}^{\ast }$ values at the second timepoint to be biased towards higher values. Meanwhile, we observed a decrease in ${T}_{2s}^{\ast }$ in this region. This suggests that if the orientation of the intra-articular grafts were the same between the two time points, a greater decrease in ${T}_{2s}^{\ast }$ would have been observed. Additionally, the femoral intra-bone grafts were closer to the magic angle than the tibial intra-bone grafts, consistent with the higher observed ${T}_{2s}^{\ast }$ values in the femoral intra-bone grafts.

One of the limitations of our study is the lack of histological confirmation of graft healing. However, obtaining histological specimen or performing arthroscopy at multiple timepoints in noncomplicated patients is impractical, and the surgeons performed systematic clinical assessments during the entire recovery period. We are also limited by the small number of patients in this preliminary study, which provides low statistical power for he observed changes. Some of the longitudinal changes as observed in this study were on the scale of measurement uncertainties and may not reflect potential physiological changes. In addition, the time points of our longitudinal analysis might be insufficient to capture dramatic changes from ligamentization in humans. An earlier first timepoint around 1 month after surgery may detect the inflammatory phase and show a more dramatic change to the fast decay ${T}_{2s}^{\ast }$ component. Furthermore, some human studies suggested that the end-point of ligamentization might be more than 1 year after surgery [40, 43] and the last time point may need to be extended. Another limitation of this study is the lack of ${T}_{2l}^{\ast }$ quantification of the ACL graft, which may behave differently than native ACL or tendon. Although results from our numerical simulations and in vivo healthy volunteers demonstrated that the introduced estimation bias would be very small for high bound water fraction, this may not be the case during the final stage of ligamentization (> 1 year). In addition, the selection of TEs in this study was experimentally determined to achieve good quality of UTE images and reliable quantification of the short T_2_* component for native ACL. Further optimization on the number of acquisitions and TE values are needed to increase the robustness of the proposed method for ACL grafts.

## Conclusion

This preliminary study has demonstrated the feasibility of applying the proposed abbreviated UTE MRI paradigm to quantitatively evaluate the fast decay ${T}_{2s}^{\ast }$ component within a clinically feasible acquisition time for ACL graft reconstruction patients (~ 8 min). We observed longitudinal changes to the short T_2_* component of the ACL grafts, potentially due to graft healing processes such as ligamentization and tendon-to-bone healing. This method has the potential for wide-spread adoption of quantitative UTE for routine clinical evaluation of the short T_2_* component of various connective tissues.

## Data Availability

Data and analysis code are available from the corresponding author upon reasonable request.

## Abbreviations

${T}_{2l}^{\ast }$: T_2_* for the slow decay component of ACL graft
${T}_{2s}^{\ast }$: T_2_* for the fast decay component of ACL graft
*f*_*bw*_: Bound water signal fraction
ACL: Anterior cruciate ligament
DTI: Diffusion tensor imaging
MRI: Magnetic resonance imaging
ROIs: Regions-of-interest
UTE: Ultrashort echo time
vTE: Variable TE
